# Are Severe Mastitis Cases in Dairy Cows Associated with Bacteremia?

**DOI:** 10.3390/ani11020410

**Published:** 2021-02-05

**Authors:** Julia Brennecke, Ulrike Falkenberg, Nicole Wente, Volker Krömker

**Affiliations:** 1 Department of Microbiology, Faculty of Mechanical and Bioprocess Engineering, University of Applied Sciences and Arts, 30453 Hanover, Germany; brenner3108@googlemail.com (J.B.); nicole.wente@hs-hannover.de (N.W.); 2 Cattle Health Service of the Mecklenburg Vorpommern Animal Disease Fund, Neustrelitzer Str. 120/C, D-17033 Neubrandenburg, Germany; u.falkenberg@tskmv.de; 3 Department of Veterinary and Animal Sciences, University of Copenhagen, 1870 Frederiksberg C, Denmark

**Keywords:** severe mastitis, bacteremia, *E. coli*

## Abstract

**Simple Summary:**

The occurrence of bacteremia associated with cases of severe mastitis in dairy cows is an under-researched topic and of great practical importance for the development of evidence-based strategies in mastitis therapy. The aim of this study was to determine the occurrence of bacteremia in lactating dairy cows with severe mastitis cases. For this purpose, we worked on the detection of culturable pathogens in the blood to obtain information on whether systemic antibiotic therapy is necessary in cases of severe mastitis. Detection of culturable pathogens in the blood of cows with severe clinical mastitis was apparently rare (1.4%). Further studies are necessary to estimate the occurrence of bacteremia in severe bovine mastitis.

**Abstract:**

The objective of this study was to investigate the occurrence of bacteremia in dairy cows with severe mastitis. Milk samples were collected from affected udder quarters, and corresponding blood samples were collected from dairy cows with severe mastitis at the time of diagnosis before any therapeutic measures were undertaken. The cultural detection of pathogens in blood classified a bacteremia. Further diagnostic tests were performed to provide evidence of bacteremia. This was realized by PCR with regard to *S. aureus, E. coli* and *S. uberis* and the Limulus test. Detection of culturable pathogens in the blood of cows with severe clinical mastitis was rare and occurred in only one of 70 (1.4%) cases. Overall, bacterial growth was detected in 53 of 70 (75.7%) milk samples. *S. uberis* (22/70), *E. coli* (12/70) and *S. aureus* (4/70) were the most frequently isolated pathogens from milk of cows with severe mastitis. PCR was performed in 38 of 70 (54.3%) blood samples. PCR was positive in eight of 38 cases. *S. uberis* was found most frequently in six blood samples (8.6%). *E. coli* was found on PCR in one blood sample (1.4%). *S. aureus* was identified in one blood sample (1.4%). When Coliforms were detected in the quarter milk sample, a Limulus test was performed in the corresponding blood sample. In three of 15 cases, the Limulus test was positive (4.3% of samples). Further studies are needed to investigate the occurrence of bacteremia in cows with severe mastitis in a higher population size.

## 1. Introduction

Mastitis is the most frequent disease in dairy cows and is recognized as having a negative impact on animal welfare and dairy farm profitability. Animal-friendly, economical, resource-saving milk production provides the basis for sustained consumer acceptance [1]. At present, antibiotic therapy is essential to balance the bovine udder health, animal welfare and economic aspects [2].

Every case of mastitis is expensive as this means a loss of milk, increased time expenditure, a shortened life span of the animals, high treatment costs, frequent use of antibiotics, and potentially, the loss of the affected udder quarter or even loss of animals [3,4,5]. Among all reasons for culling, mastitis is mentioned in five to 17% of cases [6].

Clinical mastitis can be classified as follows: Mild mastitis (M1) is characterized only by changes in secretion, while moderate mastitis (M2) additionally shows local signs of inflammation (redness, swelling, increased heat, sensitivity to pain) of the mammary gland, while dairy cows with severe mastitis (M3) also show general disorders (such as fever, low temperature, loss of appetite or inability to stand [7,8,9,10]. The highest percentage of mastitis cases are mild (M1, 50%), followed by moderate (M2, 35%) and severe (M3, 15%) cases [9]. A large recent study found 9.1% severe cases in 2883 incidents of clinical mastitis [11]. Depending on the farm structure and hygiene status, approx. 20–30% cases of udder inflammation are caused by Gram-negative microorganisms (e.g., *E. coli*, *Klebsiella* spp.) [12]. Severe mastitis cases are almost as frequently caused by Gram-positive as by Gram-negative microorganisms. In 35% of cases of severe mastitis, no pathogen growth occurred. In particular, Gram-negative pathogens could be frequently isolated from blood samples of cows with severe mastitis [11].

The mortality rate for cases of severe mastitis associated with detection of *E. coli* was 35.0% [13].

Studies focusing exclusively on severe mastitis are rare. Typical sample size of severe mastitis studies varied between as many as 104 cases in Erskine et al. [14] and 69 cases in Krömker et al. [15]. Studies are limited because of the need for rapid treatment, as severe mastitis is associated with a high risk of death. Severe mastitis is of particular importance because, in addition to a negative effect on the general condition of dairy cows and local and systemic irreparable tissue damage, it also leads to bacteremia and even death [13,16,17]. Pathogens cause cell damage in the udder, whereupon inflammation sets in as a defensive reaction of the body (e.g., swelling, redness and heat). Increased blood circulation in the affected udder quarter leads to an accelerated transport of immune cells, antibodies and an accelerated removal of cell debris. The explanation for the spreading mastitis is a reduced function and impaired defense of the neutrophil granulocytes, which leads to a faster multiplication of the bacteria [18]. Toxins or pathogens flood the body via the bloodstream and cause damage in other organs, which can lead to blood poisoning [13,19].

A differentiated consideration of the terms bacteremia and sepsis is required when defining the term “bacteremia”. While bacteremia is defined as the presence of bacteria in the blood [20], sepsis is a generalized inflammatory response, a clinical diagnosis, that requires further specification regarding the source of infection and the etiologic pathogen. [21].

A study by Wenz et al. [13] investigating the presence of microorganisms in blood samples from affected cows reported that 32% of cows with severe coli mastitis developed bacteremia.

Regardless of the pathogen, it is recommended to prevent the increased expression of the factors involved in the inflammation in order to relieve the animal from pain, suffering and damage and to stabilize its general condition [22]. This is achieved with an anti-inflammatory therapy with a non-steroidal anti-inflammatory agent in clinical mastitis, which should therefore be adopted [19]. With the 16th Amendment of the German Medicines Act, a benchmarking system was established as an instrument for antibiotic contamination reduction in livestock farming. Thus, veterinarians as well as animal owners are obliged to implement the antibiotic minimization concepts in common practice [23].

In cases of severe mastitis, it is a common doctrine in Germany that parenteral antibiotics should always be administered immediately due to the risk of bacteremia, regardless of individual animal factors and identified pathogens [13,24]. 

Nonetheless, the effect of antibiotic therapy is highly dependent on risk factors such as clinical findings, animal species-specific factors and the pathogen causing the mastitis [19]. If the pathogen is not known at the beginning of the therapy, a therapy with broad-spectrum antibiotics seems to be reasonable [19]. However, when administered systemically, these antibiotics also influence the intestinal flora and can thus lead to the development of resistance. For reasons of animal welfare, every diseased animal is entitled to appropriate treatment [25]. It would therefore be advantageous if the risk of bacteremia in cases of severe mastitis could be better assessed.

In this context, the aim of our study was to gain more information about the occurrence of bacteremia in cases of severe mastitis.

## 2. Materials and Methods 

All applicable guidelines for the care and use of animals were followed. The study was reviewed by the Animal Welfare Committee of Hanover University of Applied Sciences and Arts, Hanover, Germany. An application for a license for animal testing was not required by the local government. The study met the International Guiding Principles for Biomedical Research Involving Animals (1985).

### 2.1. Study Design 

A cross-sectional study was carried out from January 2017 to December 2017 on two commercial dairy farms in Mecklenburg-Western Pomerania, Germany. The herd size varied between 1000 and 1200 Holstein-Friesian dairy cows. The 305-d milk yield (i.e., milk quantity of the first 305 days of lactation) ranged from 11,000 to 13,202 kg, with a bulk milk somatic cell count of 164,000 to 280,000 cells/mL. The cows received a total mixed ration (TMR) depending on their production level and were milked three times a day. All study animals were housed in free-stall barns with cubicles. The farms participated in a dairy herd improvement (DHI) program.

Cases of severe mastitis were detected during routine milkings on farms by milking personnel in accordance with the IDF Standard (i.e., secretion change + udder swelling + disturbance of general condition) [26]. A milk sample from the affected quarter was collected immediately after detecting severe mastitis. The farm veterinarian took a blood sample from the affected animal under aseptic conditions. Afterwards, the sick cow was treated by the veterinarian following farm-specific therapy plans, i.e., a local and systemic antibiotic treatment, fluid therapy and NSAID.

### 2.2. Sampling

The milking personnel were trained by the study veterinarians in collecting a quarter milk sample according to the guidelines of the Society of Veterinary Medicine (GVA) [27]. After pre-milking using pre-milking cups, the teat of the affected udder quarter was disinfected using disinfectant wipes (70% ethanol), and milk samples was taken aseptically using sample tubes held horizontally and first opened immediately below the quarter. These were stored in a cool place in tubes containing boric acid as a preservative until shipment [27]. Blood was drawn from the jugular vein of the affected cow by a veterinarian. The skin was disinfected by applying povidone-iodine scrub and 70% ethanol three times above the jugular vein. A total of 15 mL of blood was collected aseptically through an 18-gauge needle and transferred to 9 mL of sterile brain-heart infusion broth (Merck KGaA, Darmstadt, Germany).

All samples were sent to the microbiology laboratory of Hannover University of Applied Sciences and Arts within two days for microbiological analysis.

### 2.3. Laboratory Procedures

#### 2.3.1. Milk Samples

Microbiological analysis of the milk samples was performed in accordance with the GVA guidelines [28]. A total of 10 µL of each milk sample was cultured on esculin blood agar (Oxoid Deutschland GmbH, Wesel, Germany). The samples were transported within two days without cooling, but using a boric acid containing preserving agent [29], to the microbiology laboratory of Hannover University of Applied Sciences and Arts for microbiological analysis. Boric acid is a preservative of mesophilic microorganisms in milk [29]. The plates were analyzed after a 24-h and 48-h incubation period at 37 °C. The grown colonies were initially differentiated by their hemolysis status, esculin hydrolysis, cell morphology and Gram staining. Non-hemolytic Gram-positive catalase positive cocci (3% H_2_O_2_, Merck KGaA) were identified as non-*aureus* staphylococci (NaS), and β hemolysis staphylococci were further differentiated using the clumping factor test (DiaMondiaL Staph Plus Kit, Sekisui Virotech GmbH, Russelsheim, Germany). *S. aureus* showed a clumping factor positive reaction and NaS were negative. Catalase-negative Gram-positive cocci, which hydrolyzed esculin were subcultivated on modified Rambach agar [30] to distinguish between *S. uberis* and Enterococcus species. Esculin non-hydrolyzing Gram-positive, catalase-negative cocci were further grouped using Lancefield serotyping (DiaMondiaL Streptococcal Extraction Kit, Sekisui Virotech GmbH) and referred to as *Streptococcus* (*S*.) *agalactiae*, *Streptococcus* (*S*.) *dysgalactiae* and *Streptococcus* (*S*.) *canis*. Gram-positive, β-hemolytic and catalase- and esculin-negative irregular rods with Y-shaped cell configuration were identified as *Trueperella* (*T*.) *pyogenes*. Gram-positive, non-hemolytic catalase-positive irregular rods were specified as coryneforms. Gram-negative rods were differentiated by their ability to catabolize glucose under aerobic and anaerobic conditions (glucose-supplemented oxidation-fermentation test medium, (Merck KGaA)), and their ability to produce cytochrome C oxidase (Bactident Oxidase, Merck KGaA). Cytochrome C oxidase negative rods fermenting glucose were cultured on Chromocult^®^ Coliform Agar (Merck KGaA) to distinguish *Escherichia* (*E*.) *coli* and other Coliforms. Non-motile Coliforms were reported as *Klebsiella* spp.; Gram-negative, cytochrome C oxidase-producing bacteria, which metabolized glucose oxidatively, were defined as *Pseudomonas* spp.; yeasts and *Prototheca* spp. were differentiated by microscopy.

The samples were declared as contaminated if more than two different colonies were identified per plate, although *S. aureus*, *S. dysgalactiae* and *T. pyogenes* isolates were taken into account.

#### 2.3.2. Blood Samples

After incubating the blood and brain heart broth mixture at 37 °C for 24 h, all blood samples were analyzed by the cultural method in accordance with GVA [27] as performed for the milk samples but using a 100 µL sample volume instead of 10 µL. If no culturable pathogens could be detected in the blood, the next step was to search for pathogen components (DNA, endotoxins in the case of Gram-negative microorganisms). Such components at least give an indication that the corresponding microorganisms were also in the blood.

#### 2.3.3. DNA Extraction and PCR

The extraction of the bacterial DNA from blood samples was performed in accordance with the DNAeasy Blood & Tissue Kit (QIAGEN Benelux B.V., Venlo, the Netherlands). The PCR was carried out in 25 µL reaction mix including 12.5 µL ReadyMix™ Taq PCR Reaction Mix (Sigma-Aldrich Chemie GmbH, Taufkirchen Munich, Germany), 20 pmol of primer (listed in Table 1), 5 µL of the template and H_2_O for no template control. Amplification reactions were performed in a Stratagene Mx3005P qPCR System Thermocycler (Agilent Technologies Inc., Santa Clara, CA, USA). The temperature profile was programed as previously described by Riffon et al. [31]. The PCR products were directly stained with Midori Green Direct (Nippon Genetics Europe GmbH, Düren, Germany) and separated in a 2% agarose gel at 100 V for 2 h.

#### 2.3.4. Limulus Test

The Limulus (limulus amoebocyte lysate) test is a test method for detecting pyrogens. The test for Gram-negative bacterial endotoxin was performed for blood samples utilizing the ToxinSensor™ Gel Clot Endotoxin Assay Kit (GenScript USA Inc., Piscataway, NJ, USA). This kit is based on Limulus Amoebocyte Lysate and the minimal detection limit of the endotoxin level occurs at 0.25 EU/mL.

## 3. Results

A total of 70 severe clinical cases of mastitis were enrolled in the study. From these cases, quarter milk samples and corresponding blood samples were examined by microbiological culture.

Overall, bacterial growth in quarter milk samples was detected in 53 of 70 (75.7%) milk samples. There was no growth in 17 (24.2%) milk samples. *S. uberis* (22/70, 31.4%), *E. coli* (12/70, 17.1%) and *S. aureus* (4/70, 5.7%) were the most frequently isolated pathogens from milk of cows with severe mastitis (Table 2).

In the blood samples, culturable pathogens were detected in one of 70 cases (*E. coli* 1.4%).

PCR was performed in 38 of 70 (54.3%) blood samples, as these had a positive bacterial culture from milk for *S. uberis*, *E. coli* and *S. aureus*. No PCR examination was performed on 32 of 70 (45.7%) blood samples as this led to exclusion if NaS, *S. dysgalactiae*, *Enterococcus* spp., Coryneform bacteria, *T. pyogenes*, other Coliforms or Yeasts were detected in the bacterial examination.

PCR was positive in eight of 38 (21.1% of PCR tests, 11.4% of cases) blood samples. *S. uberis* was found most frequently in six cases (15.8% of PCR tests, 8.6% of cases). *E. coli* was found in one of 70 (2.6% of PCR tests, 1.4% of cases). *S. aureus* was identified in 2.6% of PCR tests, 1.4% of cases (Table 2). 

The Limulus test was performed in corresponding bloods samples when *E. coli* or Coliforms were detected in quarter milk samples (15 of 70 blood samples (21.4%). The Limulus test was positive in three of 15 cases (20.0% of Limulus test results, 4.3% of cases, Table 2).

In one of 70 cases, we found culturable bacteria in the blood and in ten of 70 severe cases, pathogen components were found in the blood that may indicate bacteremia.

## 4. Discussion

Large studies focusing exclusively on severe mastitis are rare [14,15]. Certainly, the relevance of the presented data is somewhat limited, since only two, but nevertheless fairly large farms were involved. A total of 70 cases of severe mastitis could be detected during the defined period in about 2000 animals. The clinical expression of severe mastitis can vary from cow to cow [32]. This is because infection of a mammary gland depends on exposure to microorganisms, udder defense mechanisms and environmental risk factors [33], and therefore can be influenced by external factors. In addition, the mammary gland is a complex open, self-regulating system, so different symptoms may occur depending on the type and amount of pathogens and the cow’s defense behavior. In our study, most cases of clinical mastitis were caused by environmental pathogens (*S. uberis*). The same conclusion was also reached by a recent study [11].

In the present study, the occurrence of bacteremia was rare (1.4%). In contrast, based on blood culture results, Wenz et al. found that 32% of cows with acute E. coli mastitis had bacteremia [13]. The occurrence and development of bacteremia depend on pathogenicity and virulence of the pathogens. Predisposing factors, endogenous defense mechanisms, the functional state of the mammary gland tissue as well as the success of treatment form a complex interplay and determine the severity of clinical symptoms and the course of mastitis [34,35].

The connective tissue is edematous due to mastitis, and diffuse or focal redness and necrosis may occur. The frequent occurrence of thrombi in the blood and lymphatic vessels can lead to death of individual udder areas and promote the formation of sequestered udders. With the death of the udder epithelium, important defense mechanisms are lost, and the invading toxins cannot be buffered and thus enter the bloodstream. This results in bacteremia [36].

Due to the risk of impending bacteremia and consequent loss of individual animals, parenteral administering of antibiotics is generally accepted by farmers and veterinarians. Current evidence indicates that parenteral antibiotic therapy should be administered immediately after diagnosis of severe cases of mastitis because of the disruption of the general condition and the resulting risk of bacteremia [37].

Our findings do not support the regular occurrence of bacteremia in cases of severe mastitis. Pathogen components (DNA and endotoxins) can still be found in the blood, but the extent to which this should be relied upon as an indication of systemic treatment is questionable.

Following our diagnostic measures (milk sample and blood sample), local udder therapy, parenteral therapy, if required, and fluid therapy were applied, which have been demonstrated to have a positive effect on animal welfare [38]. Studies have shown that in all cases of mastitis, administering an NSAID has a positive effect on clinical and bacteriological recovery as well as on milk yield [19,39].

It is evident that this issue is of significant importance both from an animal welfare aspect and in the context of the antibiotic reduction program.

According to a human medical study, the presence of a pathogen in at least two blood cultures taken was considered definitive evidence of bacteremia [20]. The collection interval (0 and 24 h) provides better sensitivity. Performing multiple samples may reduce the risk of misinterpretation of contamination as infection.

The diagnostic sensitivity of our study is limited; primary sensitivity was restricted by the amount of blood tested (1 cfu/15 mL). We chose the amount of blood studied in accordance with the study of Wenz et al. (15 mL).

The timing of blood collection is critical, as bacteremia precedes the rise in temperature by approximately one hour [7]. Blood samples are therefore best collected before the expected temperature rise or as early as possible at the onset of fever [7,8,9,10].

In a field study, diagnosis, sampling and therapy can and must occur when the animal is presented for veterinary examination, i.e., severe mastitis was diagnosed. Thereafter, a milk sample was taken immediately, the veterinarian was informed, and the veterinarian took a blood sample at the next possible time and then treated the animal accordingly. The blood samples were taken exclusively by the same veterinarian to eliminate possible sources of error.

The aim was to determine the infection status as close as possible to the time of development of mastitis. The limiting factor was the milking interval (3× daily; 04:00–09:00, 12:00–17:00, 20:00–0:30); i.e., severe mastitis was only detected during milking times. Thus, cases of mastitis could show clinical symptoms for a maximum of eight hours. This could have an impact on the detection rate of bacteremia.

Furthermore, only sick cows with severe mastitis were considered in the present study. Future studies should be conducted to identify further differences between healthy and sick cattle. Indeed, the results of a prospective case-control study suggest that bacteremia is common in both healthy postpartum dairy cows and dairy cows with acute puerperal metritis (APM), cases of which occurred in 53% of cattle in each group [40]. 

In addition to animal selection and sampling period, the sampling interval, storage and transport of samples are also important for detecting live pathogens [21]. The site of collection and adequate preparation thereof by shaving and disinfecting are important. In general, hygienic hand disinfection must be performed before blood cultures are collected [41,42]. In addition, proper performance of skin antisepsis in the area of the puncture site has a decisive influence on the contamination rate of peripheral blood cultures. According to the results of recent studies, any approved skin antiseptic is suitable when used correctly [43,44].

Successful isolation of pathogens is directly dependent on the volume of collected blood, as low blood volume limits the sensitivity of the diagnostic agent [45]. We followed other researchers’ experience regarding specific standards in veterinary medicine [13]. 

Transporting blood samples to the laboratory for analysis is critical for pathogen isolation [45]. The milk samples in the present study were stored under refrigeration. Transport from the barn to the laboratory took five hours. Of course, some pathogens and thus evidence of bacteremia may have been lost in the process. 

In summary, the occurrence of culturable pathogens in the blood of cows with severe mastitis is rare. PCR and the Limulus test can be helpful in detecting pathogen components; culture tests provide the most reliable and unambiguous results. 

However, further investigations are necessary and must be assessed in individually cases with regard to the animals’ appearance and well-being. 

## 5. Conclusions

Severe mastitis is a life-threatening disease in dairy cows, requiring immediate action. Due to the assumption that these cases are associated with bacteremia, they are currently treated with systemic antibiotics. Contrary to expectations, in our study, bacteremia occurred very rarely in severe mastitis with typical mastitis pathogens.

Further studies on larger numbers of animals and on more farms with an even broader spectrum of pathogens are needed to more accurately estimate the occurrence of bacteremia in cases of severe mastitis.

## Figures and Tables

**Table 1:** Primer in accordance with Riffon et al. [31].

Primer	Specificity	Annealing Temperature (°C)	Sequence (5′–3′)	Size of Amplified Product (bp)
Forward Eco 223 reverse Eco 455	*E. coli*	64	ATC AAC CGA GAT TCC CCC AGT TCA CTA TCG GTC AGT CAG GAG	232
Forward Sub 154 6reverse Sub 2170	*S. uberis*	59	TGA TGG GGA GCG AAA ATA AG CCC AAC AAC GCC TCA AAC GA	624
Forward Sau 327 reverse Sau 1645	*S. aureus*	64	GGA CGA CAT TAG ACG AAT CA CGG GCA CCT ATT TTC TAT CT	1318

**Table 2:** Comparison of findings in associated milk and blood samples.

Pathogens	Cultural Evidence in Mastitis Secretion [n (%)] *	Cultural Detection in Blood [n (%)] *	Species-DNA Detection in Blood by PCR ** [n (%)] *	Positive Limulus Test *** [n (%)] *
Coliforms	1 (1.4)	0	0	1 (1.4)
*S. uberis*/*E. coli*	1 (1.4)	0/0	0/0	1 (1.4)
*E. coli*	12 (17.1)	1(1.4)	1 ** (1.4)	1 (1.4)
Coryneform bacteria	1 (1.4)	0	n.p.	n.p.
*S. dysgalactiae*	4 (5.7)	0	n.p.	n.p.
*Enterococcus* spp.	2 (2.3)	0	n.p.	n.p.
Yeasts	1 (1.4)	0	n.p.	n.p.
NaS	2 (2.3)	0	n.p.	n.p.
*S. aureus*	4 (5.7)	0	1 (1.4)	n.p.
*S. uberis*	22 (31.4)	0	6 (8.6)	n.p.
*S. uberis*/NaS	1 (1.4)	0/0	0/n.p.	n.p.
*T. pyogenes*	1 (1.4)	0	n.p.	n.p.
No growth	17 (24.2)	0	n.p.	n.p.

* of a total of 70 (100%), ** performed in cases of *S. uberis*, *E. coli* and/or *S. aureus* detection in the milk sample, *** performed in cases of Coliform identification in the corresponding milk sample, n.p.: not performed.

## Data Availability

The data presented in this study are available on request from the corresponding author. The data are not publicly available due to privacy.
